# Supplementary material for: Optimization of Rifapentine-Loaded Lipid Nanoparticles Using a Quality-by-Design Strategy
Source: Pharmaceutics. 2020 Jan 17;12(1):75. doi: 10.3390/pharmaceutics12010075 (PMC7022298; doi:10.3390/pharmaceutics12010075)
Supplement: Supplementary file 1 [file pharmaceutics-12-00075-s001.pdf]

| | Sum of squares | df | Mean square | *F*-value | *p*-value |
|---|---|---|---|---|---|
| $X_1$ | **6845.00** | **1** | **6845.000** | **30.69507** | **0.031068** |
| $X_1^2$ | 612.06 | 1 | 612.058 | 2.74465 | 0.239426 |
| $X_2$ | 1960.20 | 1 | 1960.200 | 8.79013 | 0.097423 |
| $X_2^2$ | 87.75 | 1 | 87.750 | 0.39350 | 0.594534 |
| $X_3$ | **6552.20** | **1** | **6552.200** | **29.38206** | **0.032390** |
| $X_3^2$ | 36.06 | 1 | 36.058 | 0.16169 | 0.726505 |
| $X_1X_2$ | 72.25 | 1 | 72.250 | 0.32399 | 0.626622 |
| $X_1X_3$ | 1369.00 | 1 | 1369.000 | 6.13901 | 0.131513 |
| $X_2X_3$ | 225.00 | 1 | 225.000 | 1.00897 | 0.420931 |
| **Error** | 446.00 | 2 | 223.000 | | |
| **Total SS** | 18691.73 | 14 | | | |
| $R^2$ | 0.976 | | | | |
| **Adj.**$R^2$ | 0.833 | | | | |

**Supplementary Table 1. Analysis of variance for particle size ($Y_1$).** Statistically significant parameters (*p*-value < 0.05 with a 95% confident interval) are highlighted in bold.

| | Sum of squares | df | Mean square | *F*-value | *p*-value |
|---|---|---|---|---|---|
| $X_1$ | 16.0205 | 1 | 16.0205 | 1.73695 | 0.318234 |
| $X_1^2$ | 10.7231 | 1 | 10.7231 | 1.16261 | 0.393691 |
| $X_2$ | 57.1220 | 1 | 57.1220 | 6.19321 | 0.130578 |
| $X_2^2$ | 6.9385 | 1 | 6.9385 | 0.75228 | 0.477191 |
| $X_3$ | 66.2480 | 1 | 66.2480 | 7.18265 | 0.115580 |
| $X_3^2$ | **233.6078** | **1** | **233.6078** | **25.32791** | **0.037288** |
| $X_1X_2$ | 3.2400 | 1 | 3.2400 | 0.35128 | 0.613476 |
| $X_1X_3$ | 89.3025 | 1 | 89.3025 | 9.68224 | 0.089616 |
| $X_2X_3$ | **193.2100** | **1** | **193.2100** | **20.94796** | **0.044570** |
| **Error** | 18.4467 | 2 | 9.2233 | | |
| **Total SS** | 748.9773 | 14 | | | |
| $R^2$ | 0.975 | | | | |
| **Adj.**$R^2$ | 0.828 | | | | |

**Supplementary Table 2. Analysis of variance for encapsulation efficiency (*$Y_2$*).** Statistically significant parameters (*p*-value < 0.05 with a 95% confident interval) are highlighted in bold.

| | Sum of squares | df | Mean square | $F$-value | $p$-value |
|---|---|---|---|---|---|
| $X_1$ | **0.695645** | **1** | **0.695645** | **115.3003** | **0.008562** |
| $X_1^2$ | 0.000108 | 1 | 0.000108 | 0.0180 | 0.905671 |
| $X_2$ | **0.856980** | **1** | **0.856980** | **142.0409** | **0.006967** |
| $X_2^2$ | 0.028001 | 1 | 0.028001 | 4.6410 | 0.164033 |
| $X_3$ | 0.040500 | 1 | 0.040500 | 6.7127 | 0.122247 |
| $X_3^2$ | **0.163478** | **1** | **0.163478** | **27.0957** | **0.034981** |
| $X_1X_2$ | 0.016900 | 1 | 0.016900 | 2.8011 | 0.236175 |
| $X_1X_3$ | 0.042025 | 1 | 0.042025 | 6.9655 | 0.118568 |
| $X_2X_3$ | **0.122500** | **1** | **0.122500** | **20.3039** | **0.045888** |
| **Error** | 0.012067 | 2 | 0.006033 | | |
| **Total SS** | 2.054373 | 14 | | | |
| $R^2$ | 0.994 | | | | |
| **Adj.**$R^2$ | 0.959 | | | | |

**Supplementary Table 3. Analysis of variance for loading capacity ($Y_3$).** Statistically significant parameters ($p$-value < 0.05 with a 95% confident interval) are highlighted in bold.

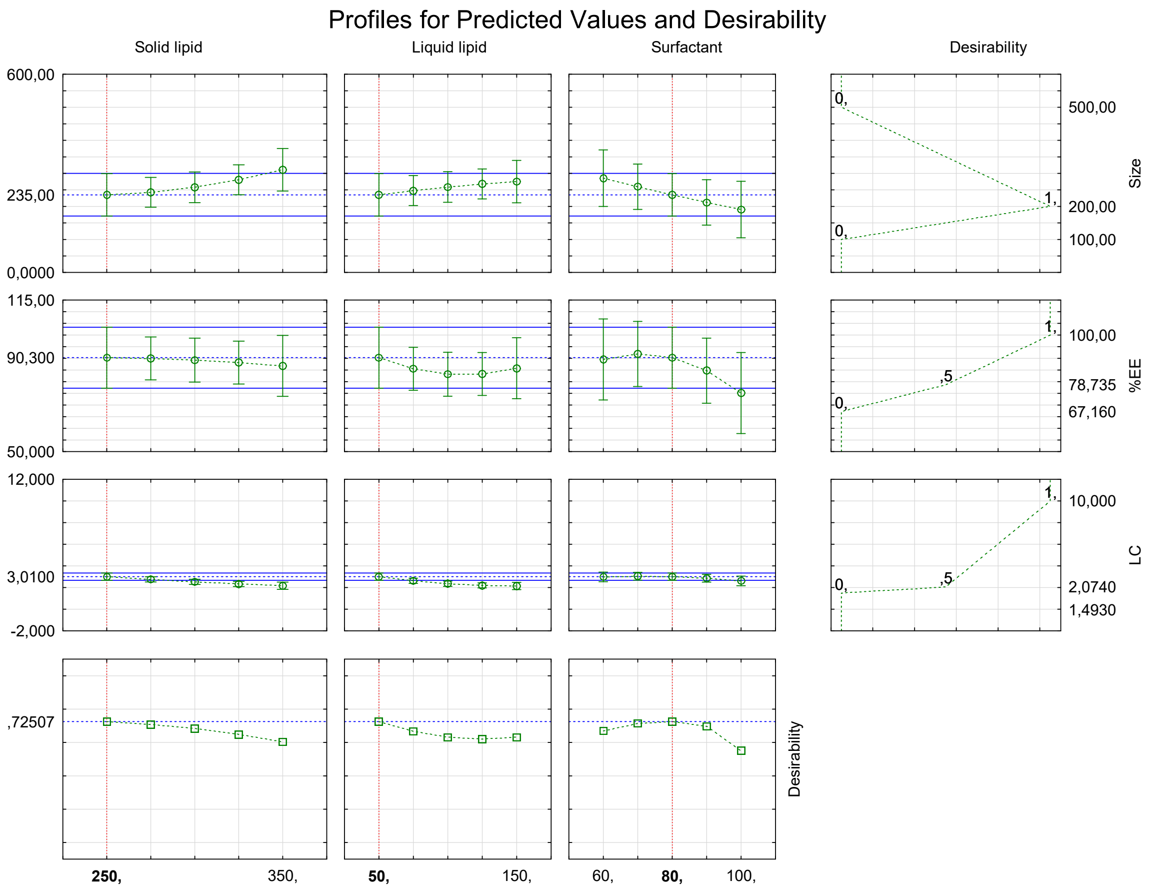


**Supplementary Figure 1. Response desirability profile of the optimized formulation.** The prediction and profiling function of STATISTICA 10 (Statsoft[1]. Inc.) software was used to obtain the response desirability profile. The factors were settled as particle size in the optimum value of 200 nm, the maximum encapsulation efficiency, and the maximum loading capacity.
